# Methicillin-resistant staphylococcal contamination of cellular phones of personnel in a veterinary teaching hospital

**DOI:** 10.1186/1756-0500-5-193

**Published:** 2012-07-10

**Authors:** Timothy Julian, Ameet Singh, Joyce Rousseau, J Scott Weese

**Affiliations:** 1 Department of Pathobiology, Ontario Veterinary College, University of Guelph, Guelph, ON, N1G 2W1, Canada; 2 Department of Clinical Studies, Ontario Veterinary College, University of Guelph, Guelph, ON, N1G 2W1, Canada

**Keywords:** Cellular phone, Methicillin-resistant staphylococcus, Veterinary, Contamination

## Abstract

**Background:**

Hospital-associated infections are an increasing cause of morbidity and mortality in veterinary patients. With the emergence of multi-drug resistant bacteria, these infections can be particularly difficult to eradicate. Sources of hospital-associated infections can include the patients own flora, medical staff and inanimate hospital objects. Cellular phones are becoming an invaluable feature of communication within hospitals, and since they are frequently handled by healthcare personnel, there may be a potential for contamination with various pathogens. The objective of this study was to determine the prevalence of contamination of cellular phones (hospital issued and personal) carried by personnel at the Ontario Veterinary College Health Sciences Centre with methicillin-resistant *Staphylococcus pseudintermedius* (MRSP) and methicillin-resistant *Staphylococcus aureus* (MRSA).

**Results:**

MRSP was isolated from 1.6% (2/123) and MRSA was isolated from 0.8% (1/123) of cellular phones. Only 21.9% (27/123) of participants in the study indicated that they routinely cleaned their cellular phone.

**Conclusions:**

Cellular phones in a veterinary teaching hospital can harbour MRSP and MRSA, two opportunistic pathogens of significant concern. While the contamination rate was low, cellular phones could represent a potential source for infection of patients as well as infection of veterinary personnel and other people that might have contact with them. Regardless of the low incidence of contamination of cellular phones found in this study, a disinfection protocol for hospital-issued and personal cellular phones used in veterinary teaching hospitals should be in place to reduce the potential of cross-contamination.

## Background

Nosocomial or hospital-associated infections (HAIs) are an increasing cause of morbidity and mortality in human and veterinary medicine [1-4]. Multi-drug resistant (MDR) bacteria are commonly implicated in HAIs and can be challenging to eliminate [1-4]. Sources of HAI can include medical staff, the patients own flora and inanimate hospital objects [5,6]. Hands of healthcare personnel are commonly contaminated with opportunistic pathogens and poor hand hygiene compliance is thought to be an important factor in the pathogenesis of HAIs [7]. Contaminated hands can result in direct transfer of pathogens to patients, as well as contamination of inanimate objects (fomites). Any items that have frequent hand contact, especially in the absence of routine hand hygiene practices, are at high risk of becoming contaminated. Cellular phones (CPs) have become an indispensable accessory of todays society and they are being used extensively in a hospital setting to optimize patient care and client communications. However, CPs are commonly handled (irrespective of the cleanliness of hands), rarely disinfected and could harbour pathogenic bacteria. Concerns regarding bacterial contamination associated with the use of CPs within the hospital environment have been raised in human medicine, and studies of human healthcare worker CPs have reported contamination of 943% of CPs with bacteria known to cause HAIs [8-18]. Comparable data are not available for veterinary medicine.

Methicillin-resistant *S. aureus* (MRSA) is a critically important hospital-associated pathogen in humans [19] and has been found on 1.910% of CPs sampled in hospitals [8-18]. It is also a significant concern in companion animals, both as a cause of HAI [1-3,20] and the potential for zoonotic transmission to veterinary personnel [21,22]. Of greater relevance from an animal health aspect is methicillin-resistant *S. pseudintermedius*, which has rapidly emerged as a leading cause of various opportunistic infections, including pyoderma [23,24] and surgical site infection [1-3].

The purpose of this study was to determine the MRSA and MRSP contamination rate of CPs used by personnel in the Ontario Veterinary College Health Sciences Centre (OVCHSC) and to identify factors associated with contamination. In this study we used electrostatic cloths to recover bacteria from CPs. This methodology has previously been described for the recovery of bacteria from inanimate objects [25-27]. This method was chosen because electrostatic cloths are easy to use, inexpensive and readily available. Furthermore, a standardized sampling technique for the recovery of bacteria from inanimate objects does not exist and the sensitivity of bacterial recovery from various techniques such as contact plates, electrostatic cloths and cotton-tipped swabs is unknown.

## Methods

### Sample collection and study population

This cross-sectional study was conducted from August to September 2011 at the OVCHSC. Hospital personnel, including veterinary students, technicians, residents/interns and clinical faculty were recruited for participation in this study. Upon verbal consent, participants CPs (hospital-issued and/or personal) were sampled and a self-administered questionnaire was completed. The questionnaire characterized the participants position in the hospital, frequency of CP cleaning, and whether the personal CP (if present) was used while in the OVCHSC. This study was approved by the University of Guelph Research Ethics Board.

### Microbiological analysis

Samples were collected by wiping an electrostatic cloth (Swiffer, Procter & Gamble, Cincinnati, OH, USA) along all surfaces of the CP using a gloved hand [25-27]. After sampling, the cloth was placed into a pre-labeled sterile bag for later inoculation. All samples were collected by the same individual (TJ) and gloves were changed between each sample. To investigate potential sources of sampling contamination, electrostatic cloth samples were also collected from the hands and forearms of the individual (TJ) performing the sampling, the box containing the gloves, the clipboard and questionnaire sheets. During each sampling period, new, unused, electrostatic cloths were removed from the box and cultured as negative controls [25].

Enrichment culture was performed by adding 7080mL of enrichment broth consisting of 10g tryptone/L, 75g sodium chloride/L, 10g mannitol/L, and 2.5g yeast extract/L to sterile bags containing electrostatic cloths immediately following sample collection. After 24h incubation at 35C, 1 loopful (approximately 10L) of broth was inoculated onto MRSA Chromogenic agar (BBL CHROMagar MRSA, Becton, Dickinson and Co., Mississauga, ON, Canada) and Mannitol Salt Agar with 2g/mL oxacillin and incubated at 35C for MRSP selection. Plates were examined after 24 and 48h of incubation.

Isolates were identified as *S. aureus* by colony morphology, pink color, Gram stain appearance, catalase reaction, coagulase reaction, and *S. aureus* latex agglutination test (Pastorex Staph-plus, Bio-Rad Laboratories Ltd, Mississauga, ON, Canada). Methicillin-resistance was confirmed by penicillin binding protein 2a latex agglutination test (MRSA latex agglutination test, Denka Seiken, USA Inc, Campbell, CA, USA). *Staphylococcus pseudintermedius* was identified by colony morphology, Gram stain appearance, catalase and coagulase reactions and a species-specific non-commercialized PCR assay [28].

### MRSA characterization

Isolates were typed by sequencing of the X region of the protein A gene (spa typing) as described [29]. For spa typing, sequences were analyzed using eGenomics software (http://tools.egenomics.com). Ridom database equivalents were identified using the Ridom Spaserver website (http://www.spaserver.ridom.de). eGenomics spa types are reported using a numerical system (i.e. spa type 539) while Ridom spa types are reported using a numerical system preceded by a t (i.e. spa t034). Real-time PCR was used to detect the *lukF* and *lukS* components of the Panton-Valentine leukocidin (PVL) using PVLSC-F (5-GCTCAGGAGATACAAG-3) and PVLSC-R (5-GGATAGCAAAAGCAATG-3) primers [30].

### MRSP characterization

MRSP isolates were characterized by sequence analysis of the *mec*-associated direct repeat unit (*dru* typing) [31], with dru repeats and types assigned by the Dru-typing.org database (http://www.dru-typing.org/search.php).

### Statistical analysis

Contamination rate data were described. Categorical associations were assessed using Fishers exact test (FET), with significance set at *P*<0.05. Statistical analysis was performed using commercially available software (InStat, GraphPad Software Inc., La Jolla, CA, USA)

## Results

One hundred and twenty three CPs from 106 individuals were sampled. Seventy-one were personal CPs while 52 were hospital-issued CPs (Table 1). Methicillin-resistant staphylococci were isolated from 3/123 (2.4%) CPs (Table 1). MRSP was isolated from two (1.6%) CPs while MRSA was isolated from one (0.8%). Both MRSP positive CPs were from samples collected on the same day and both were classified as dt9a. Samples were collected in an anonymous manner, with only identification of the personnel group (e.g. technician) so it was impossible to retrospectively investigate any potential associations between the two MRSP positive samples. Methicillin-resistant staphylococci were not isolated from any negative control samples.

**Table 1:** MRSA and MRSP contamination rate of personal and hospital-issued CPs by veterinary hospital personnel

Group	Personal phones	Hospital-issued phones	Total
Technician	2/8 (25%) (1 MRSA, 1 MRSP)	0/15	2/23 (8.7%)
Residents/Interns	0/8	1/18 (5.6%) (MRSP)	1/23 (4.3%)
Veterinary student	0/37	0	0/37
Faculty	0/10	0/13	0/23
Non-medical personnel	0/8	0/6	0/14
Total	2/71 (2.8%)	1/52 (1.9%)	3/123 (2.4%)

The MRSA isolate was identified as *spa* type 18/t338 [32]. Based on available Canadian data [32] and Ridom Spaserver website (http://www.spaserver.ridom.de), this strain was inferred to be a clonal complex 30 strain that is classified as Canadian epidemic MRSA (CMRSA)-4, USA200, and eMRSA-16. It did not contain genes encoding PVL. The MRSP isolates were identified as dru type 9a, typically associated with sequence type 71 [33].

There was no difference in contamination between different personnel classifications (*P*=0.2, FET) overall, however there was a significant difference between groups when only personal CPs were considered (*P*=0.004, FET) but not with hospital-issued phones (*P*=0.6, FET). There was no difference overall between personal and hospital-issued phones (*P*=1.0, FET).

Twenty-two percent (27/123) of sampled CPs had reportedly been disinfected in the past month. Two/27 (7.4%) of the CPs that were contaminated had reportedly been disinfected in the past month versus 1/96 (1.0%) that had not been disinfected (*P*=0.1, FET). Sixty-four of the 71 (90%) of individuals carrying personal CPs reported using them while on the OVCHSC premises.

## Discussion

Contamination of CPs was uncommon but, nonetheless, both MRSA and MRSP were identified. These results support concerns that CPs could act as a fomite for pathogenic bacteria, with transmission to patients or personnel through subsequent contamination of the hands. The relevance of CP contamination is unclear but these data, along with similar data from human medicine raise concern [8-18]. People frequently handle CPs, and likely do so irrespective of the cleanliness of their hands. Goldblatt *et al.* reported that physicians used CPs excessively, even during patient contact, which likely contributed to a higher rate of CP contamination compared with nursing staff [18]. These factors create the potential for both contamination of the phone from contaminated hands, and transfer of pathogens from a contaminated CP to clean hands. Proper hand hygiene has been emphasized as a means of reducing the incidence of nosocomial infections [7] and is probably a key factor for reducing CP contamination. If hand hygiene is properly performed before and after patient contact, and after contact with potentially contaminated environmental sites, the risk of CP contamination would be minimized. However, hand hygiene compliance rates in human medicine remains below 50% [34] and there is no evidence suggesting better compliance in veterinary healthcare personnel.

In a human healthcare study, physician CPs were more often contaminated than those of nursing staff (60% vs 20%) [18]. Here, there was no significant difference overall, however there was a significant difference between groups when only personal CPs were considered, with contamination identified only on technician CPs. Reasons for this are unclear and were not specifically investigated here. It is possible that contamination could be of greater risk in technicians because they may have more contact with animals and handle more animals overall compared to the other groups. At the OVCHSC, personnel use their personal CP in the hospital and community, whereas hospital-issued CPs are used only within hospital premises. Although there was no significant difference in contamination between personal and hospital-issued CPs, further study of this association and other factors associated with CP contamination are required.

MRSP is a significant problem in companion animals, and the presence of this multidrug opportunistic pathogen is a concern [35-38]. This is not surprising since MRSP can be found in clinically normal animals [39] and in the veterinary hospital environment [40]. Considering the significant problems encountered in treating some MRSP infections, especially implant-associated surgical site infections, measures to reduce transmission of this pathogen are important. The MRSP isolates identified here were dt9a, which is among the most common dru types in North America and typically associated with sequence type 71 [33].

MRSA is both an animal health and zoonotic concern. The isolate identified here was *spa* type 18/t338, a human epidemic clone. Typing cannot indicate the origin of MRSA because companion animals are typically infected with human MRSA clones, and no testing of personnel was performed to differentiate animal versus human sources [20].

Only 21.9% of sampled CPs had been disinfected within the past month. Method of disinfection was not queried in the survey administered to participants. Facility infection control protocols do not address disinfection of CPs and that, combined with lack of consideration of the potential for CPs to become contaminated, may explain the low disinfection rates. There is also limited information about CP disinfection methods that are both effective and do not damage the CP. However, the use of 70% isopropyl alcohol wipes eliminated bacterial contamination in 98% of mobile phones in one study [41], and this is a simple measure that could be used routinely.

## Conclusions

While uncommon, contamination of CPs with MRSA and MRSP was identified, supporting concerns that these devices may be fomites for transmission of infection to patients or personnel. Further, since personal CPs are used in the hospital and community, they represent a potential bridge between the hospital and community and could transfer zoonotic pathogens to anyone that has contact with the CP. The true risk of CP contamination is not known and whether these fomites play a role in transmission of MRSA and/or MRSP requires further investigation. Increased consideration should be given to reducing contamination, particularly avoiding handling CPs when hands might be contaminated and using good hand hygiene practices. Routine disinfection of CPs, such as with alcohol wipes, while unproven in a veterinary context, should be considered as part of a general infection control program.

## Competing interests

The authors declare that they have no competing interests.

## Authors contributions

TJ performed data collection and drafted the manuscript. TJ and JR performed microbiological testing. AS and JSW were responsible for study design and manuscript review. All authors read and approved the final manuscript.
